# *STC2* Inhibits Hepatic Lipid Synthesis and Correlates with Intramuscular Fatty Acid Composition, Body Weight and Carcass Traits in Chickens

**DOI:** 10.3390/ani14030383

**Published:** 2024-01-25

**Authors:** Yuzhu Cao, Qihui Jia, Yuxin Xing, Chenglin Ma, Hongbo Guan, Weihua Tian, Xiangtao Kang, Yadong Tian, Xiaojun Liu, Hong Li

**Affiliations:** 1 College of Animal Science and Technology, Henan Agricultural University, Zhengzhou 450046, China; caoyuzhu9891@163.com (Y.C.); jqh9702@163.com (Q.J.); xyx15754@163.com (Y.X.); 13782241532@163.com (C.M.); 18703988707@163.com (H.G.); tianweihua126@126.com (W.T.); xtkang2001@263.net (X.K.); ydtian111@163.com (Y.T.); xjliu2008@hotmail.com (X.L.); 2 International Joint Research Laboratory for Poultry Breeding of Henan, Zhengzhou 450046, China; 3 Henan Key Laboratory for Innovation and Utilization of Chicken Germplasm Resources, Zhengzhou 450046, China

**Keywords:** Stanniocalcin 2, variation, lipid metabolism, fatty acid, poultry

## Abstract

**Simple Summary:**

The effect of Scalarpin 2 (*STC2*) on chicken hepatic lipid metabolism is still unknown. In this study, we found that genetic variation rs9949205 occurring in the *STC2* was significantly associated with chicken body weight at different weeks and carcass traits. The inhibitory effect of *STC2* on lipid synthesis in LMH cells was observed, and its expression level in muscle showed a significant association with 176 lipids, predominantly enriched in essential omega-3 and omega-6 fatty acids. The evidence suggests that *STC2* involved growth and development, as well as lipid metabolism in chickens.

**Abstract:**

Stanniocalcin 2 (STC2) is a secreted glycoprotein involved in multiple biological processes. To systemically study the biological role of *STC2* in chickens, phylogenetic tree analysis and conservation analysis were conducted. Association analysis between variations in the *STC2* gene and the economic traits of Gushi-Anka F2 was conducted. The tissue expression patterns of *STC2* expression in different chicken tissues and liver at different stages were detected. The biological role of *STC2* in chicken liver was investigated through overexpression and interfering methods in the LMH cell line. Correlation analyses between *STC2* expression and lipid components were conducted. (1) The phylogenetic tree displayed that chicken STC2 is most closely related with Japanese quail and most distantly related with Xenopus tropicalis. STC2 has the same identical conserved motifs as other species. (2) rs9949205 (T > C) found in *STC2* intron was highly significantly correlated with chicken body weight at 0, 2, 4, 6, 8, 10 and 12 weeks (*p* < 0.01). Extremely significant correlations of rs9949205 with semi-evisceration weight (SEW), evisceration weight (EW), breast muscle weight (BMW), leg muscle weight (LMW), liver weight and abdominal fat weight (AFW) were revealed (*p* < 0.01). Significant associations between rs9949205 and abdominal fat percentage, liver weight rate, breast muscle weight rate and leg muscle weight rate were also found (*p* < 0.05). Individuals with TT or TC genotypes had significantly lower abdominal fat percentage and liver weight rate compared to those with the CC genotype, while their body weight and other carcass traits were higher. (3) *STC2* showed a high expression level in chicken liver tissue, which significantly increased with the progression of age (*p* < 0.05). *STC2* was observed to inhibit the content of lipid droplets, triglycerides (TG) and cholesterol (TC), as well the expression level of genes related to lipid metabolism in LMH cells. (4) Correlation analysis showed that the *STC2* gene was significantly correlated with 176 lipids in the breast muscle (*p* < 0.05) and mainly enriched in omega-3 and omega-6 unsaturated fatty acids. In conclusion, the *STC2* gene in chicken might potentially play a crucial role in chicken growth and development, as well as liver lipid metabolism and muscle lipid deposition. This study provides a scientific foundation for further investigation into the regulatory mechanism of the *STC2* gene on lipid metabolism and deposition in chicken liver.

## 1. Introduction

In chicken, the de novo biosynthesis of lipids mostly (90%) occurs in the liver [1,2,3]. After synthesis, lipids are assembled into lipoproteins and transported via the bloodstream to target tissues, where they undergo hydrolysis and release fatty acids for utilization or storage [4,5,6]. The level of lipogenesis in chicken liver and fat deposition in the muscle are crucial factors that significantly affect chicken meat quality, thereby determining the overall quality and nutritional value of the meat [7,8]. Meanwhile, chicken hepatic steatosis is a prevalent manifestation of fatty liver, accompanied by an imbalance in hepatic lipid accumulation, transportation and metabolism [9,10,11], which has a detrimental effect on the health and productivity of laying hens, resulting in economic losses to the poultry industry. Therefore, an in-depth understanding of the key genes involved in controlling lipid homeostasis of chicken can contribute to enhancing poultry health, reproductive performance and meat quality. Consequently, this will enable improvement in feed conversion efficiency (FCE) and breeding efficiency.

Stanniocalcin 2 (STC2) is a secreted glycoprotein involved in multiple biological processes [12,13,14]. Related studies have shown that STC2 is associated with glucose homeostasis and phosphorus metabolism [15,16], proliferation, invasion and metastasis of various cancers [17,18,19]. In addition, STC2 was reported to regulate feeding behavior and body weight in mice [20,21]. The protective effects of STC2 in mice on the pancreas, liver and adipose tissue have been demonstrated [22,23,24]. Zhao et al. (2018) revealed that mouse *STC2* ameliorates hepatic steatosis by activating Signal Transducer and Activator of Transcription 3 (STAT3) signaling through in vivo and in vitro assays [25]. Sarapio et al. (2019) revealed that STC2 could decrease triacylglycerol synthesis, reducing glyceride/glycerol generation from 14C-glucose, direct phosphorylation of glycerol and fatty acid synthesis from 14C-glucose in eWAT of fed rats [26]. Khal et al. (2021) also demonstrated STC2 involvement in the mammalian liver gluconeogenesis pathway [27]. Joshi et al. (2022) identified mouse *STC2*, a novel aryl hydrocarbon receptor (AhR) target gene regulated by endogenous AhR agonists and tryptophan metabolite cinnabaric acid (CA) as having a protective effect on cells with alcohol-induced liver injury [28]. Patil et al. (2023) found that CA-induced AhR-mediated STC2 induction can attenuate fatty liver degeneration, inflammation and liver injury in Non-Alcoholic Fatty Liver Disease (NAFLD) [29]. The aforementioned articles suggest that *STC2* is involved in regulating lipid metabolism in mammals and might exert a positive effect in controlling hepatic lipid metabolism homeostasis.

However, until now, few reports on the *STC2* gene have existed regarding chicken, especially regarding hepatic lipid metabolism. Mittapalli et al. (2006) cloned chicken *STC2* and found that it is expressed in developing rhabdomyosarcoma and joints [30]. Sah et al. (2018) found that the *STC2* gene is associated with eggshell mineralization and can increase alkaline phosphatase activity [31]. Action of *STC2* on lipid metabolism remains poorly understood in chicken, and our question is does *STC2* control lipid metabolism in chicken? The present study systematically investigated the evolutionary conservation, expression pattern and genetic variants of the chicken *STC2* gene, as well as its involvement in lipid metabolism. Additionally, we examined the correlation between *STC2* gene expression levels and the molecular composition of pectoral intramuscular fat. The findings of this research serve as a valuable guide and reference for further investigation into the role of *STC2* in lipid metabolism, as well as for genetic enhancement in chicken breeding.

## 2. Materials and Methods

### 2.1. Sample Collection

The experimental chickens (Lushi blue-shell hens) were provided by the germplasm resource farm of Henan Agricultural University. All chickens were caged under the same environmental conditions and had ad libitum access to feed and water, in accordance with China’s yellow-feathered broiler rearing and management technical regulations (NY/T 1871-2010) [32]. The method of euthanasia for chickens is cervical dislocation. The liver tissues of chickens at the age of 10, 20, 30, 50 and 75 weeks old were collected with eight birds in each time point. The liver, duodenum, spleen, ovary, heart, kidney, leg muscle and pectoral muscle tissues were harvested from Lushi blue-shell hens at the age of 20 weeks old. All collected samples were immediately frozen in liquid nitrogen and subsequently stored at −80 °C.

### 2.2. Bioinformatics Analysis and SNP Site Screening

The amino acid sequences of STC2 from different species were downloaded from the National Center for Biotechnology Information database (GRCg7b, NCBI: https://www.ncbi.nlm.nih.gov/, accessed on 22 September 2023). The software Molecular Evolutionary Genetics Analysis version 10.0 (MEGA10.0) was utilized to perform a comparative analysis of the amino acid sequences of STC2 across various species and construct a phylogenetic tree. The online software MEME (Version 5.5.5) (https://meme-suite.org/meme/tools/meme, accessed on 23 September 2023) was utilized to analyze the conservative motif of STC2 amino acid sequence.

DNA sequence of chicken *STC2* gene (containing promoter region 2000 bp) was downloaded from the NCBI database (GRCg6a, NCBI, such as https://www.ncbi.nlm.nih.gov/genome/gdv/browser/genome/?id=GCF_000002315.6, accessed on 24 May 2023). The SNP sites occurring in *STC2* gene were screened from the GBS database of Gushi × Anka F_2_ resource population previously published by Zhang et al. [33].

### 2.3. Phenotype Data Collection

A total of 734 chickens from Gushi × Anka F_2_ resource population generated as described previously [34] were used for association analysis. The BW traits of the F_2_ resource population at the ages of 0, 2, 4, 6, 8, 10 and 12 weeks were recorded, respectively. The carcass traits including semi-evisceration weight (SEW), evisceration weight (EW), breast muscle weight (BMW), leg muscle weight (LMW), liver weight, abdominal fat weight (AFW) and some corresponding percentages were measured after the chickens were slaughtered at 12 weeks.

The intramuscular fat (IMF) content was determined using the Soxhlet (2014) extraction method [35]. For the high groups (G43wHM) and low IMF groups (G43wLM) of the Gushi chicken, refer to the description of Wang et al. (2023) [36]. The non-targeted lipidomic data and transcriptome data of pectoral muscles from 43-week-old Gushi hens with high IMF (*n* = 8) and low IMF (*n* = 8) content were obtained from our previously reported information [37]. These Gushi chickens were housed in individual cages with a coop temperature of 25–28 °C and humidity of 40–70%; they were fed with a standard commercial corn/soybean diet and water ad libitum; after 14 weeks of age, the feed contained 12.75 MJ kg^−1^ of metabolizable energy (ME) and 15.6% crude protein (CP). The lipidomic data included 733 lipid molecules belonging to four distinct categories, namely sterol lipids, sphingolipids, glycerophospholipids and glycerides.

### 2.4. Vector Construction and Small Interfering RNA (siRNA) Synthesis

The *STC2* gene coding sequence (CDS) including *HindIII* and *EcoRI* restriction endonuclease sites was cloned. The overexpression plasmid of *STC2* gene was constructed using pcDNA3.1 vector (Invitrogen, Carlsbad, CA). The pcDNA3.1 vector was digested with the *HindIII* and *EcoRI*. The linearized pcDNA3.1 vector and *STC2* gene CDS fragments are ligated with T4 DNA ligase (NEB, Beijing, China). The primer sequences are shown in Table 1.

### 2.5. Culture and Treatment of Chicken Leghorn Male Hepatoma (LMH) Cell Line

Chicken LMH cells were purchased from the American Type Culture Collection (ATCC) (Manassas, VA, USA). LMH cells were cultured in DMEM/F12 medium containing 10% fetal bovine serum (FBS) (BI, Kibbutz, Beit Haemek, Israel), 1% penicillin G (100 U/mL) and streptomycin (100 µg/mL) (Solarbio, Beijing, China). The culture plates were placed in an incubator containing 5% CO_2_ at 37 °C. When LMH cells fusion reached 70–80%, they were transfected with pcDNA3.1 vector and *STC2* recombination vector pcDNA3.1-*STC2* using lipofectamine 3000 reagent (Invitrogen, Carlsbad, MA, USA), respectively. Meanwhile, the siRNA of *STC2* (siRNA-*STC2*) and the negative control siRNA (siRNA-NC) were transfected into LMH cells, respectively. After treated for 24 h, cells were collected to evaluate the effect of *STC2* gene on TG and TC synthesis and the expression levels of lipid-metabolism-related genes. All experiments were repeated at least three times independently.

### 2.6. RNA Exaction, cDNA Synthesis and Quantitative Real-Time PCR (qRT-PCR)

The total RNA was extracted from tissues and cells according to the instructions of Trizol kit (Vazyme, Nanjing, China). The RNA quality was detected through agarose-gel electrophoresis and NanoDrop2000 (Thermo Scientific, Wilmington, DE, USA) ultraviolet spectrophotometer, respectively, and diluted to the same concentration with RNase-free water. According to the instructions of PrimeScriptTM RT reagent Kit (Vazyme, Nanjing, China), the cDNA was synthesized and stored at −20 °C.

The qRT-PCR was carried out on a LightCycler^®^ 96 instrument using the SYBR Green method. *GAPDH* was used as the reference gene. The reaction components consisted of 5 µL 2× QuantiFast SYBR Green Master Mix, 0.5 μL each of 10 nmol·L^−1^ forward and reverse primers, 1 µL cDNA, supplemented with RNase-free water to 10 µL. The reaction procedure included pre-denaturation at 95 ℃ for 5 min, followed by 35 cycles of amplification (denatured at 95 °C for 30 s, annealed at 60 °C for 30 s, extended at 72 °C for 30 s); the final elongation was 10 min at 72 °C.

The primers were designed using the NCBI Primer-BLAST tool (https://www.ncbi.nlm.nih.gov/tools/primer-blast/, accessed on 9 May 2022) and synthesized in Beijing Tsingke Biotech Co., Ltd. Company (Beijing, China). The primers information is listed in Table 2.

### 2.7. Detection of Intracellular Triglycerides and Cholesterol

To detect the intracellular TG and TC, cells were harvested and washed twice with 1% PBS. The intracellular TG and TC were measured according to the Cell TG and T-CHO ELISA kit instructions (Applygen, Beijing, China), respectively. The total intracellular protein was determined using the BCA Protein Assay kit (Applygen, Beijing, China).

### 2.8. Oil Red O Staining

In order to analyze the accumulation of intracellular lipid droplets, Oil Red O staining was performed. The cells were washed twice with PBS and fixed in 4% paraformaldehyde solution (Solarbio, Beijing, China) for 30 min at room temperature. Then, cells were stained with Oil Red O working solution (Sigma-Aldrich, St. Louis, MO, USA). After stained for 1 h, they were washed three times with PBS and then photographed at 200× magnification. Intracellular Oil Red O was dissolved using 100% isopropyl alcohol for 5 min, and the content of lipid droplets was quantified spectrophotometrically by measuring the absorbance at 510 nm [38,39].

### 2.9. Statistical Analysis

The association analyses were determined using the generalized linear mixed model (GLM) procedure in SPSS 24.0 (IBM, Chicago, IL, USA). The models were as follows:

Model I: Y*_ijklm_* = µ + G*_i_* + H*_k_* + f*_l_* + e*_ijklm_* (1)

(2) $$\mathrm{Model}\;\mathrm{II}\text{:}\;Y_{ijklm}=\mu +G_{i}+H_{k}+f_{l}\;+b\;(W_{ijklm}\;-\bar{W})\;+\;e_{ijklm}$$

Model I was used for association analysis between SNP and growth traits. Considering the effect of body weight on carcass traits, Model II was used for association analysis between SNP and carcass traits, where carcass weight was included as a covariate. The Y*_ijklm_* was the dependent variable (individual phenotype values), µ was the observation mean, G*_i_* was the fixed effect of SNP genotype (*i* = genotypes), H*_k_* was the effect of batch (*k* = 1, 2), f*_l_* was random effect of familial effect (*l* = 1, 7), b was the regression coefficient for the carcass weight, W*_ijklm_* was the individual carcass weight and $\bar{W}$ was the average carcass weight and e*_iklm_* the random error. Multiple comparisons were conducted using Bonferroni’s correction. *p* < 0.05 was determined as significance.

The correlation analysis between the expression level of *STC2* gene and lipid contents of muscle tissue was analyzed using Pearson’s correlation. The relative expression level of mRNA was calculated using the 2^−ΔΔct^ method; the relative expression level of gene was normalized to *GAPDH*. Data were expressed as mean ± SEM. Significant difference between groups was compared using Student’s t-test and one-way ANOVA. *p* < 0.01 is considered highly significant; *p* < 0.05 is considered significant. All data were visualized using GraphPad Prism 8.0 software.

## 3. Results

### 3.1. Phylogenetic Tree Construction and Conserved Motif Analysis of Different Species of STC2

**Figure 1:**
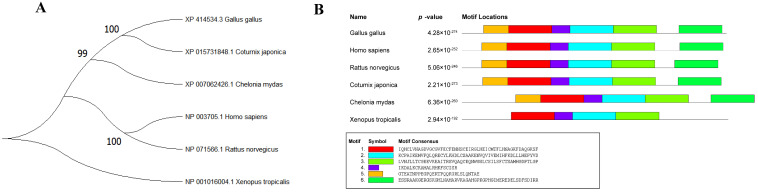
Phylogenetic tree and conservation analysis of STC2 in different species. (**A**) Phylogenetic analysis of STC2 amino acid sequences for species including chicken (*Gallus gallus*), Japanese quail (*Coturnix japonica*), green sea turtle (*Chelonia mydas*), human (*Homo sapiens*), Norway rat (*Rattus norvegicus*), tropical (*Xenopus tropicalis*). Using MEGA10.0 software, the amino acid sequences of STC2 from six species were selected and a rootless neighbor-joining phylogenetic tree was constructed, and the bootstrap test was set to repeat 2000 times. (**B**) Distribution of STC2 conserved motifs in different species. Different colored boxes indicate different conserved motif sequences.

The phylogenetic analysis revealed that STC2 of chicken and Japanese quail exhibited the closest evolutionary relationship, followed by green sea turtle, while Xenopus tropicalis was found to be the most distantly related species (Figure 1A). The conservativeness analysis revealed a high degree of conservation between chicken STC2 and the other five species, as evidenced by the presence of six identical conserved motifs (Figure 1B).

### 3.2. Association Analysis between Polymorphism in STC2 Gene and Chicken Growth and Carcass Traits

In order to understand the effect of the genetic variation that occurred in the *STC2* gene on chicken growth and carcass traits, the correlation analysis between the *STC2* gene SNP genotype and growth and carcass traits involved Gushi-Anka F_2_ population. Based on the genotyping-by-sequencing (GBS) data of F_2_ resource populations obtained from the previous report [33], nine SNPs that occurred in the *STC2* gene were detected (Supplementary Table S1). The association analysis results showed that (Table 3) rs9949205 (T > C) was highly significantly associated with chicken body weight at the ages of 0, 2, 4, 6, 8, 10 and 12 weeks, respectively (*p* < 0.01). For the carcass traits, significant correlations were observed between the rs9949205 and SEW, EW, BMW, LMW, liver weight, AFW and some corresponding percentage traits, respectively (*p* < 0.05). Except for the abdominal fat weight, abdominal fat percentage and liver weight rate, the rest of the phenotype means of individuals carrying TT or CT genotypes were significantly higher than that CC genotype individuals (*p* < 0.05). The individuals carrying the CC genotype showed significantly higher phenotype means in abdominal fat percentage and liver weight rate traits than the TT and CT genotypes (*p* < 0.05).

### 3.3. Expression Pattern of the Chicken STC2 Gene

**Figure 2:**
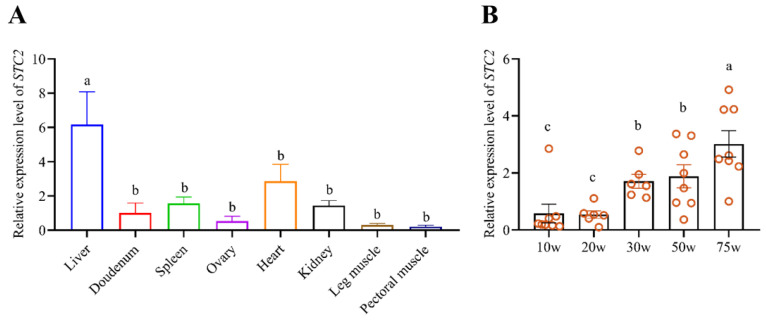
Expression characteristics of *STC2*. (**A**) Expression pattern of *STC2* in different tissues of Lushi hens at 30 weeks old (*n* = 8). (**B**) The spatio-temporal expression of *STC2* in liver tissue of Lushi chickens at different stages (*n* = 8). w means week. *n* ≥ 6 for each group. Each dot represents an individual. The relative expression of genes was normalized to *GAPDH*. Different letters mean significant difference (*p* < 0.05).

In order to understand the tissue expression characteristics, the relative expression levels of the *STC2* gene in different chicken tissues and liver at different stages were analyzed. The qRT-PCR results showed that the *STC2* gene expressed in all the detected tissues and showed the highest expression levels in liver tissue (Figure 2A). Spatio-temporal expression analysis showed that the expression level of the *STC2* gene in liver of chicken at 75w was significantly higher than that other stages (*p* < 0.05). The *STC2* expression levels in chicken livers at the ages of 30w and 50w were significantly higher than those at 10w and 20w (*p* > 0.05). No significant difference was found between 30w and 50w, and the same with 10w and 20w (*p* > 0.05) (Figure 2B).

### 3.4. STC2 Gene Inhibits the Synthesis of TG and TC in LMH Cells

**Figure 3:**
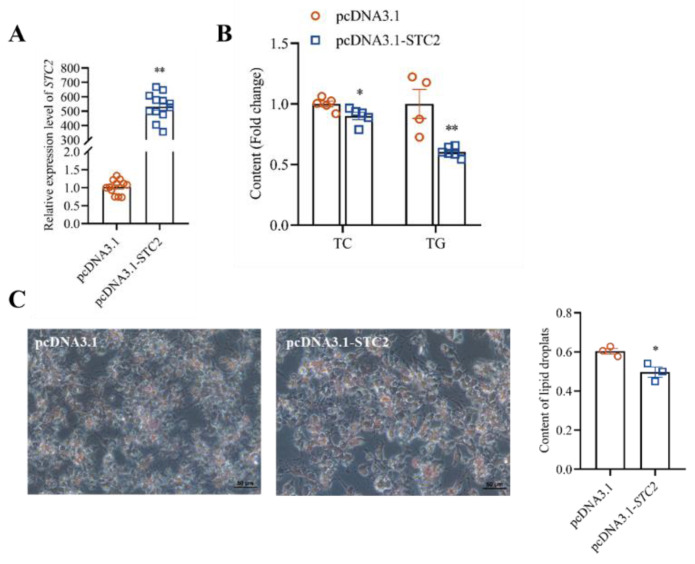
Effects of overexpression of *STC2* gene on lipid metabolism in LMH cells. (**A**) The overexpression efficiency of *STC2*. (**B**) Contents of TC and TG in LMH cells. (**C**) Lipid accumulation was evaluated with Oil Red O staining and quantified by absorbance value of the extracted Oil Red O dye. Each dot represents a repetition (*n* ≥ 4). * indicates *p* < 0.05; ** indicates *p* < 0.01.

**Figure 4:**
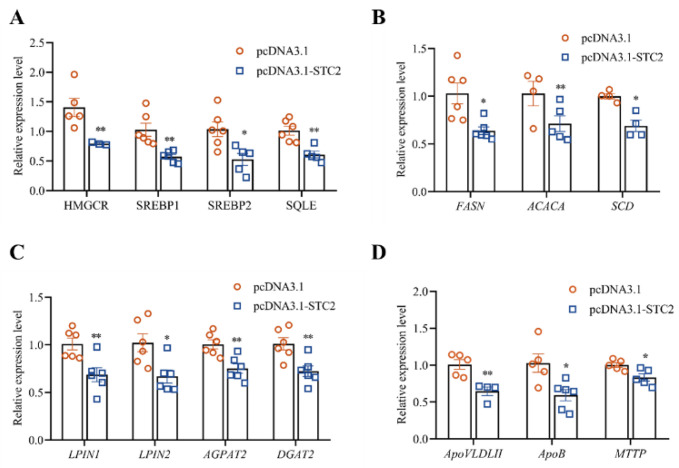
Effect of overexpression of *STC2* gene on genes related to lipid metabolism in LMH cells. (**A**) Relative expression levels of TC-synthesis-related genes. (**B**) Relative expression levels of fatty-acid-synthesis-related genes. (**C**) Relative expression levels of TG-synthesis-related genes. (**D**) Relative expression levels of lipid-transporter-related genes. Each dot represents a repetition (*n* ≥ 4). The mRNA levels of genes were normalized to *GAPDH*. * indicates *p* < 0.05; ** indicates *p* < 0.01.

To elucidate the biological function of the *STC2* gene in hepatic lipid metabolism of chicken, we transfected the STC2 overexpression vector and the control vector into LMH cells and found that there was a significant difference in the mRNA expression level of STC2 after overexpression (*p* < 0.01). At the same time, compared with the control vehicle, the contents of intracellular TC and TG were significantly decreased (*p* < 0.05) (Figure 3B). The intracellular content of lipid droplets in the *STC2* overexpressed group was significantly reduced (*p* < 0.05) (Figure 3C). The TC-synthesis-related genes, including cholesterol synthesis 3-Hydroxy-3-methylglutaryl coenzyme A reductase (*HMGCR*)*,* sterol regulatory element binding protein 1 (*SREBP1*), sterol regulatory element binding protein 2 (*SREBP2*) and squalene epoxidase (*SQLE*), were significant downregulated (Figure 4A). The fatty-acid-synthesis-related genes, including fatty acid synthesis fatty acid synthase (*FASN*)*,* acetyl-CoA carboxylase (*ACACA*) and stearoyl-CoA desaturase (*SCD*), were significant downregulated in the *STC2* overexpression group (Figure 4B). TG-synthesis-related genes, including triglyceride synthesis lipin1 (*LPIN1*)*,* phosphatidate phosphatase (*LPIN2*)*,* acylglycerol phosphate acyltransferase 2 (*AGPAT2*) and diacylglycerol O-Acyltransferase 2 (*DGAT2*), were significant downregulated (Figure 4C). Lipid-transport-related genes, including very-low-density apolipoprotein II (*ApoVLDLII*)*,* apolipoprotein B (*ApoB*) and microsomal triglyceride transfer protein (*MTTP*), were significantly downregulated (*p* < 0.05) (Figure 4D).

**Figure 5:**
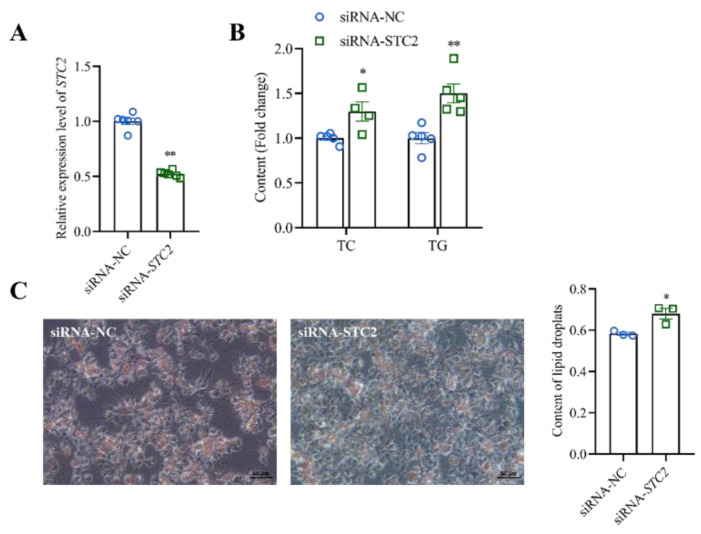
Effects of interfering with *STC2* gene on lipid metabolism in LMH cells. (**A**) Interference with *STC2* gene efficiency assay. (**B**) Contents of TC and TG in LMH cells. (**C**) Lipid accumulation was evaluated with Oil Red O staining and quantified by absorbance value of the extracted Oil Red O dye. Each dot represents a repetition (*n* ≥ 4). * indicates *p* < 0.05; ** indicates *p* < 0.01.

**Figure 6:**
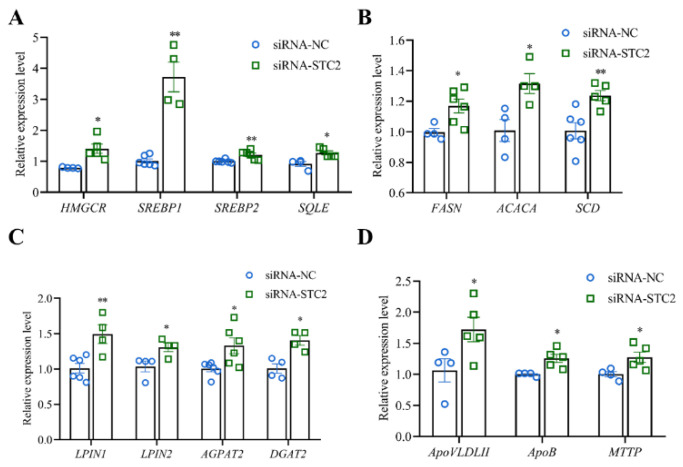
Effects of interfering with *STC2* gene on genes related to lipid metabolism in LMH cells. (**A**) Relative expression levels of TC-synthesis-related genes. (**B**) Relative expression levels of fatty-acid-synthesis-related genes. (**C**) Relative expression levels of TG-synthesis-related genes. (**D**) Relative expression levels of lipid-transporter-related genes. Each dot represents a repetition (*n* ≥ 4). The mRNA levels of genes were normalized to *GAPDH*. * indicates *p* < 0.05; ** indicates *p* < 0.01.

When LMH cells were treated using siRNA-*STC2*, the lipid droplet content (Figure 5B), the contents of TC and TG (Figure 5C) and the relative expressions of lipid-metabolism-related genes, including *HMGCR*, *SREBP1, SREBP2*, *SQLE*, *FASN*, *ACACA*, *SCD*, *LPIN1*, *LPIN2*, *AGPAT2*, *DGAT2*, *ApoVLDLII*, *ApoB* and *MTTP,* were significantly increased compared with the control group (siRNA-NC), respectively (*p* < 0.05) (Figure 6).

### 3.5. Correlation Analysis of STC2 Gene Expression with Lipid Molecules

**Figure 7:**
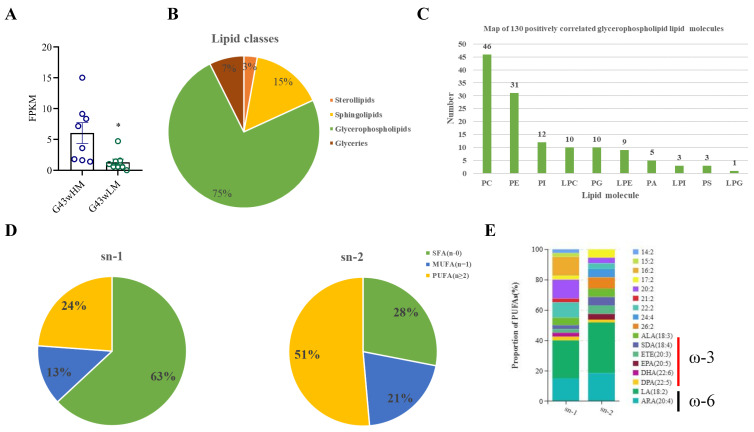
*STC2* expression contributes to the long-chain unsaturated glycerophospholipids deposition in intramuscular fat of Gushi chicken. (**A**) Transcriptomic data of the *STC2* gene in the 43-week high and low intramuscular adiposity group. G43wHM (*n* = 8) indicates high intramuscular fat group; G43wLM (*n* = 8) indicates low intramuscular fat group. (**B**) Lipid molecules in intramuscular fat significantly correlated with *STC2* expression in pectoralis of Gushi chicken. (**C**) Map of 130 positively correlated glycerophospholipid lipid molecules. (**D**) Proportion of different types of fatty acids at sn-1 and sn-2 positions of the positively correlated glycerophospholipid molecules. SFA (*n* = 0) indicates saturated fatty acids, MUFA (*n* = 1) indicates monounsaturated fatty acids and PUFA (*n* ≥ 2) indicates polyunsaturated fatty acids. (**E**) Proportional stacking of different types of MUFAs at sn-1 and sn-2 positions in (**D**). * indicates *p* < 0.05.

To further investigate the effect of the *STC2* gene on different types of lipids and fatty acids component in the pectoral muscle, we evaluated the relationship between *STC2* gene expression levels and corresponding lipid molecules in the pectoral muscle of Gushi hens at 43 weeks old (Figure 7). The results showed that the mRNA level of the *STC2* gene was higher in the high intramuscular fat group than in the low intramuscular fat group of 43-week-old Gushi chickens (*p* < 0.05) (Figure 7A). The expression level of the *STC2* gene was significantly correlated with 176 lipid molecules (*p* < 0.05). The above lipid molecules were categorized into four groups, including sterol lipids, sphingolipids, glycerophospholipids and glycerides, and glycerophospholipids exhibited the highest abundance (131/176) (Figure 7B). Further analysis showed that the expression levels of the *STC2* gene were mainly positively correlated with 130 lipid molecules in glycerophospholipids, most of which belonged to Phosphatidylcholine (PC), Phosphatidyl ethanolamine (PE), Phospholipids inositol (PI), Lysophosphatidylcholine (LPC), Phosphatidyl glycerol (PG) and Lysophospholipid ethanolamine (LPE) (Figure 7C). Specific lipid molecules are shown in Supplementary Table S2.

Among the 130 positively correlated lipid molecules, the proportions of unsaturated fatty acids in the sn-1 and sn-2 positions were 37% and 72% (Figure 7D), respectively. These polyunsaturated glycerophospholipid molecules were mainly enriched in the essential omega-3 and omega-6 fatty acids, which have beneficial effects on body health (Figure 7E).

## 4. Discussion

Phylogenetic trees can reflect the affinities and evolutionary history among different species [40,41]. In this study, we constructed evolutionary trees for the amino acid sequences encoded by the STC2 gene in different species and found that chicken STC2 has the closest affinity with birds and may have a closer common ancestor. Conservativeness is to some extent reflected in the similarity of its functions [42], and the chicken STC2 protein sequence is highly conserved among different species and may have similar biological functions.

In recent years, many studies have resolved the genetic mechanisms behind genetic variation that shape phenotypic diversity [43,44]. Jepsen et al. (2015) found that *STC2* inhibited mammalian growth by proteolytic inhibition of the insulin-like growth factor axis, and *STC2* (C120A), which cannot inhibit PAPP-A, grows like wild-type mice [45]. Marouli et al. (2017) found that genetic variation in the *STC2* gene was associated with increased height in humans through genome-wide association analysis [46,47]. Cordero et al. (2019) validated the finding that the *STC2* gene is a regulator of myogenesis by analyzing GWAS data from humans and mice [48]. In contrast, the present study investigated for the first time the variation in the STC2 gene and its biological role in lipid metabolism in chickens. According to the GBS data of Gushi-Anka and the F_2_ resource population previously published by Zhang et al. (2021) [33], the C > T mutation (rs9949205) in the *STC2* gene was screened to be significantly related with chicken BW 0, 2, 4, 6, 8, 10, 12 and carcass traits.

Individuals with TT and CT genotypes had significantly higher body weights and carcass weights than individuals with the CC genotype. Conservativeness analysis showed that chicken and mouse have a certain degree of conservativeness, and these five similar conserved motifs may be the main protein sequences for their functions. It is possible that the mutation caused changes in gene expression levels, which in turn affected body weight. Therefore, it is conjectured that the mutant TT genotype and CC genotype of the chicken *STC2* gene may have different effects on the individual growth phenotype, which needs to be further verified.

In mammals, liver and adipose tissue serve as the main sites of lipid synthesis; e.g., in pigs, fatty acid synthesis occurs mainly in adipose tissue, whereas, in chickens, liver tissue is the major organ for lipid de novo synthesis [49,50,51,52]. Excessive fat deposition reduces feed remuneration and restricts the development of the poultry industry to a certain extent [53]. We found that the *STC2* gene was specifically highly expressed in chicken liver tissues. It is known that more than 90% of de novo synthesis of fatty acids is synthesized in chicken liver [2,54,55], suggesting that the *STC2* gene may be involved in lipid metabolism.

In this study, we found that overexpression of *STC2* significantly reduced the content of lipid-metabolism-related genes, with a decrease in the accumulation of lipid droplets as well as a significant decrease in the content of TC and TG in LMH cells. Knockdown of the *STC2* gene showed the opposite results to that of overexpression. These results validated that the *STC2* gene could inhibit the lipid metabolism process in chicken LMH cells. Previous studies have shown that, in mice, the *STC2* gene alleviates cellular TG accumulation by inhibiting the lipid de novo synthesis pathway [25]. The results have been consistent with our earlier guesses. Ma et al. (2020) found that overexpression of STC2 significantly reduced lipid droplet formation in human mesenchymal stem cells (hMSC) and led to a significant decrease in peroxisome-proliferator-activated receptor γ (*PPARγ*) and fatty-acid-binding protein 4 (*FABP4*) expression [56], which is consistent with the results of the present study. The relative expression levels of STC2 in liver were significantly increased with increased chicken ages. It is well known that lipid deposition in the liver increases with the age of laying hens [57,58]. The *STC2* gene was reported to ameliorate hepatic steatosis by activating STAT3 signaling in mice [25]. Fatty liver syndrome (FLS) in chickens involves disorders of nutrient metabolism, impaired liver function and abnormal immune regulation; disorders of hepatic metabolism in chickens affect the normal functions of the liver, such as fatty acid metabolism, cholesterol synthesis, blood glucose regulation and drug metabolism [59]. The above results indicate that *STC2* might also exert a similar role in chicken hepatic steatosis, while the possible regulation pathway needs to be deeply investigated further.

Humans and mammals cannot synthesize large amounts of polyunsaturated fatty acids; they must be introduced from the diet. Among them, ω-3 and ω-6 polyunsaturated fatty acids cannot be synthesized by the human body de novo, which is more important. [60,61]. In the present study, we found that the expression levels of the *STC2* gene in pectoral muscle are significant correlated with 176 lipid molecules, which mainly belonged to PC, PE, PI, PG, LPC and LPE. The sn-2 position of one of the glycerophospholipid lipids was replaced by a large number of ω-3 and ω-6 long-chain polyunsaturated fatty acids. In recent years, it has been found that ω-3 provides certain benefits for laying hens, broilers and consumers [62,63]. Glycerophospholipids are the most abundant class of phospholipids in the body and play an important role in the study of human cardiovascular metabolic disease risk [64]. In addition, omega-3 fatty acids at the sn-2 position contain eicosapentaenoic acid (EPA), docosapentaenoic acid (DPA) and docosahexaenoic acid (DHA), which act as inflammation antagonists in favor of lowering the risk of cancer and improving the mood of the population, and an increase in the proportion of these fatty acids is beneficial to human health [65]. The ω-6 fatty acids at the sn-2 position contain linoleic acid (LA) and arachidonic acid (ARA), which have contributed significantly to the development of anti-inflammatory drugs as precursors of pro-inflammatory mediators [66], although the relative expression level of *STC2* is quite low in pectoral muscle tissue. Stanniocalcin-2 exhibits both paracrine and autocrine effects in mammalian cells [26]. It might influence the lipid type of pectoral muscle via a paracrine manner. Given the above, we found that STC2 genes might be involved in chicken growth, lipid biosynthesis and deposition in muscle through different action mechanisms.

## 5. Conclusions

In summary, we demonstrated for the first time that SNP rs9949205 in the *STC2* gene was significantly associated with chicken body weight at different stages and carcass traits, as well as the abdominal fat deposition in chickens. Moreover, individuals with TT or TC genotypes had significantly lower abdominal fat percentage and liver weight rate but higher body weight and related carcass traits compared to those with the CC genotype. This implies that SNP rs9949205 might serve as a molecular marker for marker-assisted selection in chicken breeding. *STC2* was highly expressed in chicken liver tissues and significantly increased with the progression of age. It was observed that *STC2* could suppress the synthesis of TC and TG content through decreasing the expression of genes related with lipid synthesis in LMH cells. The correlation analysis showed that the *STC2* gene may benefit the meat quality via influencing the ratio of long-chain unsaturated fatty acids and glycerophospholipid molecules enriched in chicken pectoral muscle. Our findings suggest that *STC2* might affect chicken growth, IMF and abdominal fat deposition through controlling the lipid metabolism of liver in chicken. However, further research on the biological function of the *STC2* gene in chicken growth and lipid metabolism is required.

## Figures and Tables

**Table 1:** Primers for PCR.

Gene Name	Genebank ID	Sequence (5′ → 3′)	Product Size (bp)	Tm (°C)
*STC2*	XM_414534.8	F: ctagcgtttaaacttaagctt- ATGTGCGCGGGGCTCCGC	963	60
		R: tgctggatatctgcagaattc- TCACAGAACGCAGCTAGACCTCC		

Note: lowercase indicates added homology arms including restriction sites and protective bases. The bold black are enzyme cleavage sites, and the rest are protective bases.

**Table 2:** Primers for qRT-PCR.

Gene Name	Genebank ID	Sequence (5′ → 3′)	Product Size (bp)	Tm (°C)
*STC2*	XM_414534.8	F: CGGCTGTCCCTGCAGAACACA	112	60
		R: AGCCTCGGATCTCACAAGAGT		
*HMGCR*	XM_046934671.1	F: GCGAGGAGTGTCTATTCGCA	167	60
		R: ATAGTGGTCCTGCTACGCCT		
*SREBP1*	XM_046900546.1	F: TTCTTCGTGGACGGGGATTG	218	60
		R: AGCTGAAGGTACTCCAACGC		
*SREBP2*	XM_040660556.2	F: CACCTGTGGAACAGCCTCAA	164	60
		R: GGTGAGGCATGGTAGGTCTC		
*SQLE*	NM_001194927.2	F: CATCATGGGTCTCCGAAGGG	152	60
		R: GCGGTGCATGAAGTTCCTTA		
*FASN*	NM_205155.4	F: AGAGGCTTTGAAGCTCGGAC	127	60
		R: GGTGCCTGAATACTTGGGCT		
*ACACA*	XM_046929960.1	F: GCCTCCGAGAACCCAA	128	60
		R: CCAGCAGTCTGAGCCACTA		
*SCD*	NM_204890.2	F: CAAGTTCTCCGAGACGCATG	178	60
		R: GGGCTTGTAGTATCTCCGCT		
*LPIN1*	XM_004935771.2	F: TAATGAGAGACAAGATGCCC	164	60
		R: ATCTTTTATTCTGTTTGCCAT		
*LPIN2*	NM_001006386.3	F: CCACATCTCCAATACCCACT	122	60
		R: AGTCTCTGTTTCCATAGCAT		
*AGPAT2*	XM_001235299.4	F: CACCGTCAAGAACATGAGGA	165	60
		R: ACCTCCATCAGCCCCATCAT		
*DGAT2*	XM_040661934.2	F: ACTCCAAGCCCATCACCACT	149	60
		R: CAACCCGAACCTGCCTTTGT		
*ApoVLDLII*	NM_205483.4	F: CAGGGCATTGGTGATAGCTG	162	60
		R: CCAGCTCTAGGGGACACC		
*ApoB*	NM_001044633.2	F: ATGTTCAAAAGATGCGGCCC	224	60
		R: GCATGGCTCTTCTCTCACTG		
*MTTP*	NM_001109784.3	F: CAGGAGGGATGGAGTTCAGC	166	60
		R: TGGTCACGGAATGCCTGAAA		
*GAPDH*	NM_204305.2	F: AGAACATCATCCCAGCGT	182	60
		R: AGCCTTCACTACCCTCTTG		

Note: F means upstream primer; R means downstream primer. *GAPDH* used as internal reference gene.

**Table 3:** The association analysis between genetic variant rs9949205 of *STC2* gene and economic traits in F_2_ resource population.

Traits	Mean ± SD			*p*-Value
	CC (*n* = 393)	CT (*n* = 239)	TT (*n* = 87)	
Birth weight (g)	30.391 ± 0.139 ^b^	31.223 ± 0.178 ^a^	31.157 ± 0.295 ^a^	4.56 × 10^−4^
2-week weight (g)	119.717 ± 0.934 ^b^	126.224 ± 1.165 ^a^	128.158 ± 2.013 ^a^	2.00 × 10^−6^
4-week weight (g)	312.854 ± 2.283 ^b^	333.460 ± 2.882 ^a^	336.756 ± 4.835 ^a^	2.32 × 10^−9^
6-week weight (g)	541.944 ± 4.293 ^b^	583.958 ± 5.422 ^a^	596.016 ± 9.063 ^a^	1.16 × 10^−11^
8-week weight (g)	791.321 ± 6.497 ^b^	850.956 ± 8.113 ^a^	836.937 ± 13.762 ^a^	3.53 × 10^−8^
10-week weight (g)	1084.821 ± 8.054 ^b^	1145.940 ± 10.227 ^a^	1148.57 ± 16.947 ^a^	2.00 × 10^−6^
12-week weight (g)	1321.621 ± 9.633 ^b^	1387.644 ± 12.315 ^a^	1401.965 ± 20.904 ^a^	7.00 × 10^−6^
Carcass weight rate (%)	89.581 ± 0.101	90.017 ± 0.129	90.032 ± 0.221	1.45 × 10^−2^
Weight after shedding (g)	1157.940 ± 8.480 ^b^	1222.700 ± 10.871 ^a^	1235.754 ± 18.029 ^a^	4.71 × 10^−7^
Semievisceration weight (g)	1070.511 ± 8.260 ^b^	1131.991 ± 10.611 ^a^	1145.031 ± 17.465 ^a^	8.67 × 10^−7^
Semievisceration weight rate (%)	81.086 ± 0.101 ^b^	81.700 ± 0.130 ^a^	81.767 ± 0.217 ^a^	1.60 × 10^−4^
Evisceration weight (g)	891.641 ± 7.127 ^b^	949.339 ± 9.154 ^a^	959.403 ± 15.099 ^a^	1.05 × 10^−7^
Evisceration weight rate (%)	67.556 ± 0.099 ^b^	68.415 ± 0.128 ^a^	68.468 ± 0.214 ^a^	4.30 × 10^−8^
Abdominal fat weight (g)	8.980 ± 0.585	7.153 ± 0.748	6.008 ± 1.244	3.65 × 10^−2^
Abdominal fat percentage (%)	1.006 ± 0.061 ^a^	0.735 ± 0.079 ^b^	0.625 ± 0.130 ^b^	3.56 × 10^−3^
Liver weight rate (%)	2.212 ± 0.016 ^a^	2.081 ± 0.020 ^b^	2.060 ± 0.034 ^b^	5.97 × 10^−8^
breast muscle weight (g)	67.778 ± 0.746 ^b^	73.787 ± 0.956 ^a^	72.929 ± 1.578 ^a^	1.00 × 10^−6^
breast muscle weight rate (%)	15.084 ± 0.090 ^b^	15.443 ± 0.117 ^a^	15.124 ± 0.190 ^a^	4.70 × 10^−2^
Leg muscle weight (g)	96.008 ± 0.871 ^b^	103.653 ± 1.104 ^a^	104.117 ± 1.821 ^a^	1.76 × 10^−8^
Leg muscle weight rate (%)	21.375 ± 0.077 ^b^	21.737 ± 0.099 ^a^	21.637 ± 0.161 ^a^	1.24 × 10^−2^

Note: *n* represents the number of different genotype individuals. Values with different superscripts in the line indicate significant difference *p* < 0.05. Values with same superscript in the line indicate not significant difference *p* > 0.05.

## Data Availability

The data presented in this study are available in article and Supplementary Material.

## Supplementary Materials

The following supporting information can be downloaded at https://www.mdpi.com/article/10.3390/ani14030383/s1, Supplementary Table S1: Information of SNPs location on *STC2*; Supplementary Table S2: List of lipids in intramuscular fat correlated with *STC2* expression in pectorals.
